# The Effects of College Education on Depressive Symptoms: An Instrument Variable Approach

**DOI:** 10.1155/2024/4110906

**Published:** 2024-07-11

**Authors:** Yanshang Wang, Ping He

**Affiliations:** ^1^School of Public Health, Peking University, 38 Xue Yuan Road, Haidian District, Beijing 100191, China; ^2^China Center for Health Development Studies, Peking University, 38 Xue Yuan Road, Haidian District, Beijing 100191, China

## Abstract

**Introduction:**

The acquisition of a college education is typically indicative of a resource advantage. However, in recent years, college graduates have faced increasing mental health related issues. The health advantages derived from this resource advantage have become increasingly less pronounced. This study aims to examine the effects of college education on depressive symptoms.

**Materials and Methods:**

We used data from China Family Panel Studies (CFPS), and combined this dataset with Chinese Education Examination Yearbook. We took advantage of variations in educational attainment, which was generated by college expansion policy, and adopted instrumental variables (IV) approach to identify the causal relationship.

**Results:**

Our findings indicated that college education did not have a causal effect on promoting mental health. The results were supported by the fact that they held within each subgroup. Notably, our limited evidence suggested that college expansion policy promoted equity in educational access.

**Conclusion:**

This study provided new and valuable evidence of education-induced health inequalities from the top of the education distribution.

## 1. Introduction

College education is regarded as a crucial stage in one's educational journey and is deemed more important for health than other education periods. On the one hand, college education represents a transition from student to adulthood and from adolescence to maturity, which is very consequential for one's occupation, resource, and social connection [1]. The economic loss and intensive competition in the labor market created a demand for higher educational achievement that compelled graduates to confront significant mental pressure and experience feelings of depression. The question remains, Do the benefits of college education translate into improved mental health outcomes?

Based on Grossman's [2] theory, numerous studies have examined the relationship between education and a wide range of health outcomes, but there is considerable disagreement regarding the causality [3, 4, 5]. In the literature, estimates vary greatly in size and sign, depending on the identification strategies, measurements of health, country contexts, time periods, and subpopulations employed (e.g., [6, 7, 8]). Several review studies have also reached different conclusions. The first meta-analysis study by Furnée et al. [9] found that education has a significant impact on self-reported health. The conclusion of Galama et al. [10] is that there is no convincing evidence that education influences health behaviors, although some effects are observed in specific contexts. According to the latest review by Xue et al. [11], education does not appear to have a discernible impact on health after publication bias has been corrected. It is generally believed that education is beneficial to mental health, but causal evidence is scarce and inconsistent. According to some “effect-related” studies, education improves mental health [12, 13, 14]; however, some recent studies indicate no beneficial effects or protective effects of education on mental health [15, 16, 17].

The mental health among college students is a growing concern, with a range of psychological problems becoming increasingly prominent [18]. College education represents the most significant transformation in socioeconomic status (SES) [19]. A college degree is increasingly viewed as a sign of lifestyle, class, and social origin, as well as an indication of an individual's mental disposition [20, 21]. Moreover, higher education is viewed as a resource that an individual possesses that provides benefits that can contribute to mental health [22]. On the other hand, due to the expansion of college education system, it has not only accelerated the accumulation of human capital but also the pressure from various aspects is increasing, such as elevated levels of academic and employment stress [23]. Research consistently shows a high prevalence of depression among college students, with rates ranging from 10.54% to 64.9% [24, 25]. This is further exacerbated by the increasing number of students seeking treatment for previously undiagnosed mental health issues. Therefore, in spite of the relationship between mental health and collegiate experience has been documented [26, 27], higher education is the answer to mental health inequalities remains unclear.

Research on the causality of college education on mental health is scarce. The majority of previous work has centered around low level of education due to the fact that compulsory schooling laws (CSL) are generally considered to be a part of primary and secondary education [28, 29]. A twin-pair difference-in-difference design is employed by McFarland and Wagner [14] to examine the causal effects of college on depressive symptoms and find that college education is inversely associated with depression symptoms. To the best of our knowledge, this is the first study that really identified the causal effect of college education on mental health. However, the sample in their study is limited to twins and may not reflect the demographic characteristics of the general population. A few studies have attempted to explore the relationship [19, 30]; however, all of these are “association-related” studies.

We make two main contributions to the existing literature in this study. First, it focuses on tertiary education, which is unique compared to previous studies based on CSL. This paper will contribute scarce and valuable evidence to the education-health literature, which is at the top of the education spectrum. Second, it is the first study to explore the causal effects of college education on mental health in LMICs. In our literature review, and in those by Mazumder [31], Galama et al. [10], and Xue et al. [11], no studies have provided compelling causal estimates about the impact of college education on mental health or depressive symptoms in LMICs.

In this study, we leverage a nationally representative dataset (China Family Panel Studies, CFPS), Chinese Education Examination Yearbook and China Statistical Yearbook to estimate causal effect of college education on depressive symptoms by instrument variable (IV) identification strategy.

### 1.1. Institutional Background

**Figure 1:**
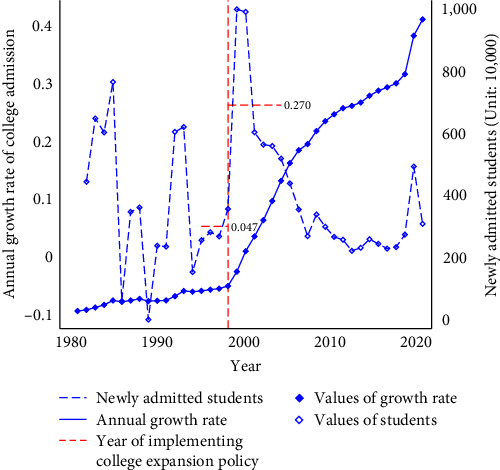
Annual growth rate of college admission and newly admitted college students in China, 1981–2020.

Since 1949, the central government has played a key role in shaping the development of higher education in China [32]. From 1949 to 1977, the number of students enrolled in colleges remained low, at less than 300,000. However, higher education has grown in scale in China since 1978. From 1981 to 1996, there was a notable increase in the number of students enrolled in college from 0.28 million to 0.97 million, as shown in Figure 1. During the 1997 Asian financial crisis, there was a temporary economic recession and rise in unemployment [32]. Despite this, the period from 1995 to 1998 saw a modest growth in college admissions, averaging 4.7% annually, due to the anticipated demand for skilled labor as a result of economic growth. Against this backdrop, in 1999, the Ministry of Education (MOE) announced the Action Plan for the Revitalization of Education in the twenty-first century, which proposed that by 2010, “the scale of higher education would be greatly expanded and the enrollment rate would be close to 15%,” and since then, there has been a rapid increase in the number of students attending colleges [33, 34]. From 1998 to 1999, college admissions in China sharply rose by 43% (Figure 1), and the average growth rate was 27% between 1999 and 2005. As shown in Figure 1, the number of newly admitted college students in China increased by 800% over a period of roughly two decades (from 1.08 million to 9.67 million).

## 2. Materials and Methods

### 2.1. Data

Our analysis relies on two main sources of data. First, the primary sample for our study is drawn from the 2012, 2016, and 2018 waves of China Family Panel Studies (CFPS), a nationally representative longitudinal study. Only these specific waves (2012, 2016, and 2018) feature data employing the Center for Epidemiologic Studies Depression Scale (CES-D8) instrument for assessing depressive symptoms. CFPS includes retrospective data on family background and early-life residence, which provides us with information regarding early-life family SES and residence information at the time of taking college entrance exam. To minimize the impact of cohort differences, we have limited the sample to individuals aged 25–40. Additionally, we limited individuals' educational attainment to high school or above. This was primarily driven by the following: to investigate the net effect of college education, we have to minimize differences in individual characteristics between the two groups (the “control group” and the “treatment group”) to reduce selection bias. Before 2000 in China, due to the imperfect education system, there were significant disparities in the duration of education received across different regions and populations. Consequently, there was no clear threshold of the age at which individuals attended college [35], making it impossible for us to divide the sample based on the age of college education.

Second, 1985–2021 Chinese Education Examination Yearbook and China Statistical Yearbook provide macrolevel data such as college enrollments and education quality. The specific ways in which these two datasets are employed are discussed in the statistical analysis section.

### 2.2. Measures

#### 2.2.1. Depressive Symptoms

The CES-D8 is used to measure depressive symptoms. Item responses were assessed on a four-point scale, where 0 indicated “rarely or none,” 1 indicated “some days,” 2 indicated “occasionally,” and 3 indicated “most of the time.” All scores specific to each question are summed to generate the depressive score (CES-D8), which ranged from 0 to 24. A higher score indicated a greater severity of depressive symptoms and behaviors [36, 37]. The items comprising the CES-D8 can be found in Table 1.

### 2.3. Statistical Analysis

As with the characteristics of this literature, endogeneity is a common problem in identifying the causal effect. Our study also faces similar identification challenges, which include endogenous bias caused by reverse causality and omitted variables, among others. Our sample construction process partially eliminates the sample selection problem. In order to further eliminate estimation bias, we use an instrument variable (IV) approach as our identification strategy.

#### 2.3.1. The First Stage

The first-stage effect of IV on college attainment is estimated by fitting:

(1) $$\begin{array}{c} {\text{College}}_{i}=\gamma_{0}+\gamma {\text{ Potential Expansion}}_{ps}+\alpha X_{i,b,p}+\delta_{b}+\theta_{p}+\theta_{p}\times \delta_{b}+{ɛ}_{i,b,p}, \end{array}$$

where *γ* is the first-stage estimate, *s* is the individual's potential year or real year for the college entrance examination, College_*i*_ is a dummy equal to 1 for individuals with any college degree or above, and *X*_*i*,*b*,*j*_ is a vector of controls that potentially vary across individuals and families. In terms of individual characteristics, we control for individual's gender and residence (Hukou12) where individual is at the age of 12. To eliminate sample selection bias, we add parental education attainment as controls. The purpose of choosing this limited set of controls is to avoid introducing bad controls. Therefore, all variables included are predetermined before obtaining college education.

*δ*_*b*_ and *θ*_*p*_ indicate birth cohort and province fixed effects, note that the province here is the province where individual was at the age of 12. As control, we include province-specific linear trend in birth cohort, denoted by *θ*_*p*_ × *δ*_*b*_, which accounts for local cohort-varying factors that may affect both health status and educational attainment. Standard errors are clustered at the province × cohort level.

CFPS provides us with the year of obtaining the highest education degree of the individual, based on this information, we calculate the potential year for the individual to take college entrance examination.

Potential Expansion_*ps*_ is the instrumental variable we constructed, and it is based on a supply-side view. Following Li et al.'s [38] study, the specific construction method is as follows:

(2) $$\begin{array}{c} {\text{Potential Expansion}}_{ps}={\text{Total Expansion}}_{s}\times {\text{Enrollment Share}}_{p,1998}, \end{array}$$

where Total Expansion_*s*_ represents the difference between actual national enrollment and its pre-1999 linear trend in year_*s*_, and Enrollment Share_*p*,1998_ represents the share of per Province_*p*_ national enrollment in 1998. The data used in the IV calculation are from the Chinese Education Examination Yearbook and China Statistical Yearbook.

As opposed to actual college enrollment expansion, Potential Expansion_*ps*_ only dependent on the latent enrollment capacity of a province. In this case, each province expands its college enrollment in accordance with its predetermined level.

#### 2.3.2. Second-Stage Model

Second-stage model can be written as follows:

(3) $$\begin{array}{c} y_{i,b,p}=\beta_{0}+\tilde{\beta }{\hat{\text{ College}}}_{i}+\delta X_{i,b,j}+\gamma_{w}+\delta_{b}+\theta_{p}+\theta_{p}\times \delta_{b}+\varepsilon_{i,b,p}, \end{array}$$

where *y*_*i*,*b*,*p*_ is CES-D8 scores of individuals *i*. The parameter of interest is $\tilde{\beta }$, which represents the average causal effect of college education. *γ*_*w*_ is the survey year fixed effects, which controls for factors changing each wave (or survey year) that are common to all individuals for a given wave.

The exclusion restriction must be satisfied in order to comply with our IV strategy. Due to the fact that the expansion policy directly affects college enrollment, and our IV is derived from the potential growth in college enrollment, we consider this assumption to be plausible.

Notably, a potential caveat is that we need to know individuals' province of taking college entrance examination (age 18), as we require to merge it with province-level instrumental variable data. According to the literature, this is a common problem, which may contribute to measurement errors as well as endogenous mobility concerns if the information of the current province is used [29, 39]. Fortunately, CFPS provides the individual's residence information at the age of 12, which is employed as a proxy for their province information of taking the college entrance examination. Therefore, it must be acknowledged that our identification strategy relies on the assumption that there has been no change in province of residence between the ages of 12 and 18.

**Figure 2:**
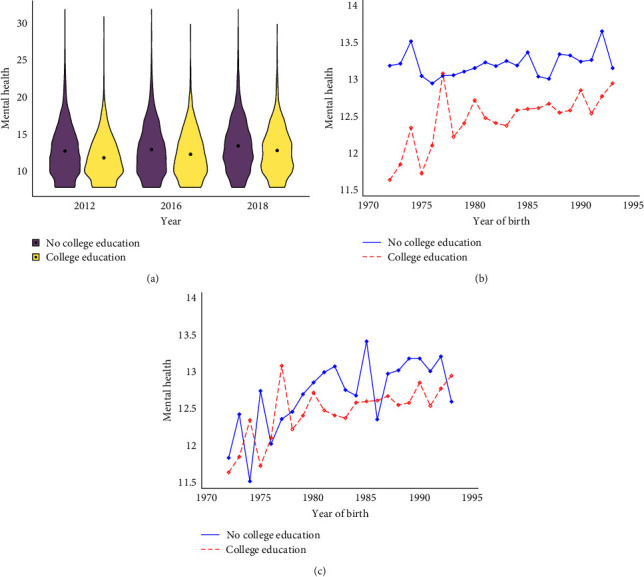
The differences of CES-D8 scores by education attainment: (a) the cross-sectional differences, (b) the full population, and (c) those with high school education and above.

Table 2 gives a summary overview of study sample by education attainment. As shown in Table 2, individuals with college education had significantly lower CES-D8 scores than those without college education. A graphical analysis of the CES-D8 is presented in Figure 2.

To further eliminate cohort effects, Figure 2(b) presents the mean values of the CES-D8 by education attainment and cohort. The mean of CES-D8 is significantly higher among those individuals with no college education, and the gap is relatively stable at about four points. Notably, when we restrict the sample to those with high school education and above, we find that this gap narrowed, albeit slightly (Figure 2(c)).

## 3. Results

### 3.1. The Association between Depressive Symptoms and College Education

This section first reports OLS estimates. The estimation results presented in the Panel A in Table 3 show that college education is associated with a more favorable mental health, and these differences are always significant.

The Panel B of Table 3 shows results when we restrict the sample to be with high school degree and above. The coefficients have the same sign as those in the entire sample. As compared with the full sample, the magnitudes of the estimates decline considerably, and 5% of the estimates are significantly different from zero. The negative association between college education and depressive symptoms has been reduced to 0.21.

These estimates should be viewed as associations rather than causal relationships, as it is not possible to control for all relevant heterogeneity between individuals with and without a college degree. Nevertheless, the implication from this result is clear: There is a strong statistically educational gradients in depression symptoms in a low-middle income country undergoing rapid social and economic development.

### 3.2. Identifying Causal Relationships

Obtaining estimates of the implied effect of college education will allow us to compare the overall associations between college education and depressive symptoms.

Tables 4 and 5 show the results of IV. We run the first-stage regressions separately for survey year (Table 4).

In the analysis of the impact of college expansion policy on education, the findings indicate a robust and statistically significant positive effect. The F-statistic exceeding 10 suggests that college expansion policy plays a pivotal role in enhancing educational attainment. Specifically, the results demonstrate that the implementation of such policy is associated with a notable increase of three percentage points in the likelihood of obtaining a college degree.

Turning to the second stage, it does not find evidence supporting a causal effect of college education on depressive symptoms. Notably, the IV estimates present a stark departure from the OLS estimates, revealing a range of values that fail to attain statistical significance in both pooled and separate samples. The range of IV estimates, spanning from −0.381 to 0.351, accompanied by standard errors ranging from 0.481 to 1.232, signifies a notable degree of variability in the estimated effects.

### 3.3. Heterogeneity Analysis

#### 3.3.1. Gender and Residence

**Figure 3:**
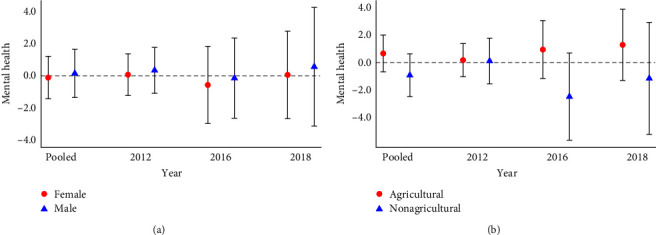
The effect of college education on mental health (grouped by (a) gender and (b) Hukou12).

To begin, we present results grouped by gender and Hukou12, since they are expected to be affected by different health processes. The results grouped by gender (Figure 3(a)) again show that college education does not have a statistically significant effect on depressive symptoms. It is worth noting that, although we also do not find heterogeneity of the effect in Hukou12 (Figure 3(b)), from the point estimates, individuals with agricultural hukou and individuals with nonagricultural hukou show opposite results.

#### 3.3.2. Family Background

Second, we examine whether the effects differ by family SES. We use father's education attainment level as a proxy. This is grounded in established theoretical frameworks that posit family SES as a key determinant of college attendance. Particularly in the context of China, where pursuing higher education often entails substantial financial investments from families, understanding the differential effects of college expansion policy across socioeconomic strata becomes imperative. The magnitude of first-stage estimates is larger for those from low-family SES (Panel A in Table 6).

#### 3.3.3. Region

We divide China's provinces into three categories based on their economic development level, i.e., east, middle, and west, and estimate the effects separately. Despite the fact that the IV estimates remain statistically insignificant in the overall results (as indicated in Table 7), we observe that the effect of the policy is stronger in the western region (as depicted in Panel B of Table 6). This highlights the policy's contribution to promoting education equity to some extent. Furthermore, we divide all provinces into low- and high-education quality provinces according to the median proportion (0.027) of people who received higher education in 1998. This stratification acknowledges the complexity of educational quality, influenced by socioeconomic and institutional factors. By doing so, we investigate if the policy's effects vary across provinces with different educational infrastructure and historical attainment levels. We make the same estimations (Panel C in Table 6), and the findings are consistent with our conjecture.

Overall, the results from the heterogeneity analyses are qualitatively similar to the findings of the overall sample, although some unexpected conclusions are also obtained.

### 3.4. Robustness Checks

We adopt a difference-in-difference (DID) strategy for intention-to-treat (ITT) effects of the expansion policy to check the robustness of our results. Although the expansion policy was implemented nationally in 1999, each province was affected to a different extent, which provides us with the opportunity to identify the causal effect of the policy.

(4) $$\begin{array}{c} y_{i,b,p}=\beta_{0}+\beta \left({\text{Intensity}}_{p}\times {\text{Born}}_{b}\right)+\gamma_{w}+\delta_{b}+\theta_{p}+\theta_{p}\times \delta_{b}+\varepsilon_{i,b,p}, \end{array}$$

where Intensity_*p*_ denotes the extent to which *p* province is affected by the expansion policy. Born_*b*_ is a dummy equal to 1 for individuals born in 1981 and later, otherwise equal to 0. Because the college expansion policy occurred in 1999, college entrance exams are generally taken at age 18.

The coefficient in front of the interaction term Intensity_*p*_ × Born_*b*_ *β* is the quantity of interest. We can interpret *β* as the average differences of CES-D8 between policy-affected cohorts and policy-unaffected cohorts, in places with a higher intensity of expansion than in places with a lower intensity of expansion. In other words, *β* captures the differences in depressive symptoms between individuals who take the college entrance examination before and after expansion policy implementation between provinces with different expansion intensity.

**Figure 4:**
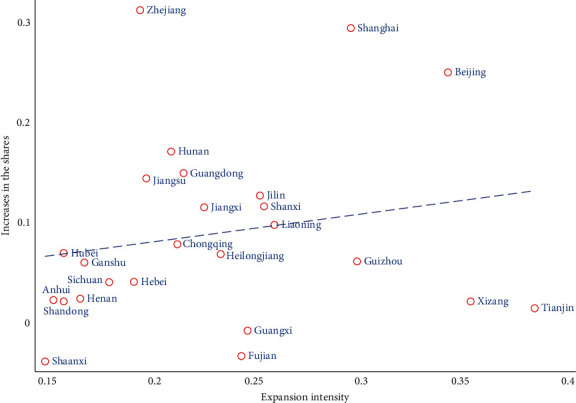
Relationship between expansion and the increase in the share of population with college and advanced degrees in each province.

Referring to Ge and Huang's [40] study, we use the number of students in local colleges divided by the number of students in high school in 1998 to measure the degree of expansion in this province. In order to verify the rationality, we calculated the proportion of people born near 1981 (born in 1981–1987 and 1974–1979) in each province with a college degree or above by using CFPS. And we calculated the difference before and after, which is the growth rate. We observe a strong and positive correlation between the growth rate and intensity across China (Figure 4).

Table 8 presents DID estimates in Equation (4). The estimates imply no effect of the expansion policy on depression symptoms: the coefficient is not significantly different from 0.

## 4. Discussion

Gradients of mental health in educational attainment have remained remarkable robust and persistent over time [41, 42]. It is often argued that these gradients are the result of causal or selection processes. Through a rigorous quantitative analysis, we contribute to addressing this question by examining the effect of college education on individuals' depressive symptoms.

We document that people have better mental health and lower depressive symptoms when they have college education. Although these differences become smaller when the population is restricted to individuals with a high school degree or higher, they remain significant. To estimate the causal effects, we exploit the college expansion policy that took place in 1999. We utilize IV strategy to estimate effect of college education, and our results show no causally protective or detrimental effect of college education on mental health. The general results are supported by the fact that they hold within each subgroup under study. Notably, although we find no causal effects, our limited evidence suggests that college expansion policies promote equity in educational access, both at the individual and regional levels.

Causally, college education does not appear to increase or diminish the risk of experiencing depressive symptoms. On one hand, numerous studies consistently demonstrate a positive correlation between higher levels of education and improved mental health [14], which is consistent with our correlation analysis results. However, on the other hand, college students, in particular, are susceptible to mental health issues, with depression being a prevalent concern [43]. Since our research focus on individuals who have completed college education rather than current college students, our findings offer insights into the long-term effects of higher education. For a long time, college education has represented a resource advantage, but with the rapid expansion of the high education, college degrees have gradually depreciated in value, and with them have come various pressures. The question of whether this resource advantage still can translate into health advantages, particularly mental health, requires prompt answers from a research and policy perspective. In this study, despite not identifying causally cumulative benefits associated with higher education, our study contributes to understanding the broader implications of college attainment on various outcomes.

Although rigorous comparisons cannot be made, we still need to review the findings of other similar causal studies. Our findings align with the conclusions of some quasiexperimental studies focusing on lower education levels [44, 45], but differ from some previous research [46]. However, their research design has the primary limitation of resulting in a small change in educational attainment that usually occurs at a lower education level. Notably, our findings are in conflict with the results of McFarland and Wagner [14]. Our study is the first study from LMICs. Furthermore, we focus on the general population, these results have better external validity. In addition, the age structure of our sample is different from their study.

The heterogenous findings shed light on the potential impact of higher education expansion policy on educational equity. Specifically, we observe that the influence of the expansion policy on college enrollment is particularly pronounced among families with lower SES and in regions characterized by lower educational quality. This underscores the policy's potential to address disparities in educational access and opportunity across different demographic and regional contexts. In addition, regarding the impact of college education on depression, divergent results among individuals of different Hukou are found, albeit statistically insignificant. These findings resonate with current trends in China and partly reflect the influence of population migration. Individuals with agricultural Hukou predominantly originate from rural areas, yet upon completing their college education, many relocate to urban areas. However, compared to their urban counterparts (non-agricultural Hukou), they often encounter heightened pressures due to resource limitations [47, 48].

While we find no casual effects, we still attempt to seek potential explanations for our findings. First, macrosocial environment plays a key role in determining the relationship between college education and mental health [14]. College education provides access to increased opportunities and resources, but in contemporary China, there has been a rapid increase in college enrollment and growth in the number of college graduates. This has resulted in significant pressure for employment, work, and life for college graduates. This is even more pronounced for individuals from rural areas, as confirmed by our heterogeneity analysis results. Second, we can gain some insight from the cumulative (dis)advantages hypothesis. Although individuals with lower education levels are more susceptible to adverse health consequences due to the lack of monetary and information resources [15], public health and social security systems may help to offset these disadvantages. The sample mean age in our study is 32.08. Based on the cumulative (dis)advantages hypothesis, health disparities between individuals with different educational levels become apparent later in life and gradually widen as they age. Scholars have reported evidence supporting this hypothesis when studying other health outcomes [49].

## Figures and Tables

**Table 1:** The items of the CES-D8.

Number	Items
N1	I am in a low spirit
N2	I find it difficult to do anything
N3	I cannot sleep well
N4	I feel happy
N5	I feel lonely
N6	I have a happy life
N7	I feel sad
N8	I feel that I cannot continue with my life

**Table 2:** Descriptive statistics.

Variable	Without college education			With college education		
	Obs	Mean	SD	Obs	Mean	SD
CES-D8	19,358	13.19	3.56	5,569	12.57	3.18
Gender						
Female	22,520	0.49	0.5	6,105	0.49	0.5
Male	22,520	0.51	0.5	6,105	0.51	0.5
Hukou12						
Agricultural	18,708	0.92	0.27	4,877	0.64	0.48
Nonagricultural	18,708	0.08	0.27	4,877	0.36	0.48
Fa Edu Attain						
Middle school or below	20,240	0.89	0.32	5,310	0.68	0.47
High school	20,240	0.1	0.31	5,310	0.24	0.43
College or above	20,240	0.01	0.09	5,310	0.08	0.27
Mo Edu Attain						
Middle school or below	20,395	0.96	0.2	5,343	0.79	0.41
High school	20,395	0.04	0.19	5,343	0.17	0.38
College or above	20,395	0	0.04	5,343	0.04	0.19

Fa Edu Attain: Father Educational Attainment; Mo Edu Attain: Mother Educational Attainment.

**Table 3:** Association of college education and depression symptoms.

Variable	Depressive symptoms		
	(1)	(2)	(3)
Panel A: full population			
Treat	−0.424*⁣*^*∗∗∗*^	−0.393*⁣*^*∗∗∗*^	−0.404*⁣*^*∗∗∗*^
	(0.079)	(0.082)	(0.085)
Mean	13.002	13	12.982
Observations	15,351	15,279	14,722
Panel B: high school or above			
Treat	−0.199*⁣*^*∗*^	−0.203*⁣*^*∗*^	−0.206*⁣*^*∗*^
	(0.094)	(0.095)	(0.098)
Individual controls			
Gender	No	Yes	Yes
Hukou12	No	Yes	Yes
Family background			
Fa Edu Attain	No	No	Yes
Mo Edu Attain	No	No	Yes
Survey year	Yes	Yes	Yes
Province	Yes	Yes	Yes
Birth year	Yes	Yes	Yes
Birth year × province	Yes	Yes	Yes
Mean	12.625	12.626	12.599
Observations	5,638	5,631	5,506

*Notes*. Standard errors clustered within province-cohort cells in parentheses. *⁣*^*∗*^, *⁣*^*∗∗∗*^Indicate significance at the 5% and 0.1% levels, respectively. Fa Edu Attain: Father Educational Attainment; Mo Edu Attain: Mother Educational Attainment.

**Table 4:** First-stage estimates.

Sample	*γ*	SE	CD Wald F	KP Wald F	Observations
Panel A					
Pooled sample	0.031	0.002	293.569	199.144	5,506
Panel B					
2012	0.036	0.002	166.29	246.562	1,802
2016	0.029	0.003	75.107	91.680	1,756
2018	0.027	0.003	54.123	71.840	1,948

*Notes*. Standard errors clustered within province-cohort cells in parentheses. It has controlled for all the aforementioned covariates.

**Table 5:** The effects of college education mental health.

Sample	*β*	SE	Mean	Observations
Panel A				
Pooled sample	0.073	0.531	12.600	5,506
Panel B				
2012	0.215	0.481	12.087	1,802
2016	−0.381	0.888	12.532	1,756
2018	0.351	1.232	13.134	1,948

*Notes*. Standard errors clustered within province-cohort cells in parentheses. It has controlled for all the aforementioned covariates.

**Table 6:** First-stage estimates (heterogeneity analysis).

Sample	*γ*	SE	CD Wald F	KP Wald F	Observations
Panel A					
Middle school or below	0.035	0.003	240.682	128.254	3,959
High school or above	0.024	0.003	58.548	52.741	1,547
Panel B					
East	0.032	0.003	177.343	146.138	2,643
Central	0.03	0.004	86.965	56.871	1,785
West	0.037	0.01	29.011	14.51	1,078
Panel C					
Low quality	0.033	0.004	122.986	77.88	2,683
High quality	0.03	0.003	170.042	127.055	2,823

*Notes*. Standard errors clustered within province-cohort cells in parentheses. It has controlled for all the aforementioned covariates.

**Table 7:** The effects of college education mental health (heterogeneity analysis).

Sample	*β*	SE	Mean	Observations
Panel A				
Middle school or below	−0.588	(0.544)	12.575	3,959
High school or above	1.485	(1.308)	12.663	1,547
Panel B				
East	0.38	(0.744)	12.275	2,643
Central	0.223	(0.833)	12.747	1,785
West	−1.793	(1.536)	13.149	1,078
Panel C				
Low quality	−0.566	(0.683)	12.858	2,683
High quality	0.541	(0.790)	12.354	2,823

*Notes*. Standard errors clustered within province-cohort cells in parentheses. It has controlled for all the aforementioned covariates.

**Table 8:** The effects of college expansion policy on depression symptoms, with DID estimates.

Variable	Mental health		
	(1)	(2)	(3)
DID	−4.111	−3.796	−3.715
	(3.459)	(3.455)	(3.447)
Individual controls	No	Yes	Yes
Family background	No	No	Yes
Survey year	Yes	Yes	Yes
Province	Yes	Yes	Yes
Birth year	Yes	Yes	Yes
Birth year × province	Yes	Yes	Yes
Mean	12.642	12.644	12.622
Observations	5,855	5,846	5,681

*Notes*. Standard errors clustered within province-cohort cells in parentheses.

## Data Availability

Data supporting this research are available upon request.

## Additional Points

*Limitations*. Our study has two limitations, which should be noted. First, data limitations prevent us from determining the exact reason why college education had no effect on depressive symptoms. Second, although to some extent that our results revealed that the college expansion policy promoted equal access to higher education, we fail to examine the impact of educational quality on health. Given that there may be trade-offs between the quantity and quality of college education, further research into the impact of educational quality on health is necessary to gain a comprehensive understanding of the relationship between college education and health. Our results highlight some important lessons for policy. First, our findings suggest that college expansion policy may contribute to promoting equity in educational access. Therefore, policymakers should prioritize investing in initiatives aimed at increasing access to higher education, particularly for marginalized populations. Additionally, integrating mental health support services into educational institutions is crucial, given the negative correlation between college education and depressive symptoms. There is a pressing need for careful and ongoing consideration of the relationship between college education and mental health, and we propose three promising research avenues going forward. First, the effects of college education on depressive symptoms will be over the long-term, or at a later age, need to be further investigated. Second, future research should also aim to further develop underlying structural and conceptual model that explain confounders and their interaction, such as genetic endowments and environmental factors [45]. Considering that college education is closely related to the macroenvironment, it is necessary to further explore the heterogeneity of the effects under different periods, economic conditions, and social structures. Finally, understanding the reasons for the nonrelationship between college education and depressive symptoms is essential to human capital theory. The in-depth analysis of causal mediation can shed light on underlying mechanisms.
